# IN MEMORIAM: John A. Sweeney, Ph.D.

**DOI:** 10.1093/psyrad/kkad032

**Published:** 2023-12-18

**Authors:** Su Lui, Xiaoqi Huang, Qiyong Gong

**Affiliations:** Department of Radiology and Huaxi MR Research Center (HMRRC), Functional and Molecular Imaging Key Laboratory of Sichuan Province, West China Hospital, Sichuan University, Chengdu 610041, China; Department of Radiology and Huaxi MR Research Center (HMRRC), Functional and Molecular Imaging Key Laboratory of Sichuan Province, West China Hospital, Sichuan University, Chengdu 610041, China; Department of Radiology and Huaxi MR Research Center (HMRRC), Functional and Molecular Imaging Key Laboratory of Sichuan Province, West China Hospital, Sichuan University, Chengdu 610041, China

Professor John Sweeney, an Associate Editor of *Psychoradiology* and Visiting Professor of the West China Hospital of Sichuan University, left us unexpectedly on September 10, 2023, at the age of 71, shortly after a visit to Chengdu, China, when having a reunion with his friends here. It is with great sadness that we remember the extraordinary contributions of the late Professor Sweeney, who made lasting impacts in the field. His dedication and expertise significantly influenced the growth and quality of the journal, leaving an indelible mark on the intersection of psychology and radiology. Professor Sweeney's commitment to advancing knowledge and fostering collaboration within the discipline will be sorely missed, but his legacy will continue to inspire future generations of scholars and practitioners in psychoradiology.

Whereas the extensive academic achievements made by John considerably exceed any simple description and compliment, his impact on the science and lives of people in the Huaxi MR Research Center (HMRRC) of West China Hospital, Sichuan University has left an indelible mark. The collaboration between John and the HMRRC started in 2008 at the annual meeting of International Society for Magnetic Resonance in Medicine (ISMRM), where he had shown tremendous interest in the drug-naïve first episode schizophrenia project we were presenting. After this, John started to work with us on neuroimaging studies on not only schizophrenia, but also other psychiatric disorders including major depressive disorder, obsessive-compulsive disorder, and post-traumatic stress disorder.

The initial collaboration and paper editing for the journal was through email exchanges, but then in 2013 John and his wife Marilynn came to Chengdu for the first time to meet all the people he had been working with on papers, many of whom were new faces. He was gentle, polite, and humble to everyone, even though he was a major leader in this field by that time. When he was visiting Huaxi, we had the opportunity to show him how we were working and the way we were conducting research. He had stayed for a week, and during his stay he traveled to many different places and tried many kinds of tea and spicy food here, all of which turned out to be his favorite. These were important reasons to bring him here to work as an advisor and visiting professor at the West China Hospital of Sichuan University in Chengdu in 2015, which was a really important, happy, and educational experience greatly treasured by everybody working here.

We consider ourselves fortunate to have had John with us in our spacious laboratory, where everyone could easily interact. Having him nearby was comforting, as we knew we could turn to him for help and guidance regarding our projects and papers. He was exceptionally generous with his time and energy, patiently addressing each student's questions and often providing in-depth explanations spanning entire paragraphs to underscore the importance of even the smallest detail. His teachings extended beyond writing and scientific methodologies, drawing from his life experiences. He had a penchant for engaging with the younger generation. Many young students held him in high respect, treating him like a close family member, often inviting him to witness their significant life events. Throughout his annual 3-month stay, we enjoyed many joyful moments, and on his departure for the USA, there was a long line waiting to host him for sumptuous meals, a tradition also observed in Chengdu.

From the academic perspective, work with him had always been productive and there are nearly 120 publications with his name listed from HMRRC. Above all, his most important work was dedicated to projects focused on schizophrenia. With his help and guidance, researchers from HMRRC have systematically characterized the anatomical and functional brain changes at the illness onset and their progressive trajectories without antipsychotic medication confounds, and longitudinal brain changes after short- and long-term antipsychotic medication. Additionally, potential biological subtypes of patients with schizophrenia have been derived from neuroimaging metrics and epigenetic measures, along with their multi-phenotyping biological correlates, and they are still under active investigation. Along the way, he guided and inspired dozens of individuals here, and some of them have become the leaders of different centers or departments. He deeply influenced the careers of many through his comforting wisdom and guidance.

John also helped build connections between the USA and China. He had been a leading PI on a NSFC-NIH joint grant; moreover, he introduced the Bipolar and Schizophrenia Network for Intermediate Phenotypes (B-SNIP) consortium and the team from the Department of Psychiatry and Behavioral Neuroscience, University of Cincinnati College of Medicine led by Melissa DelBello to HMRRC. His efforts to promote USA–China collaborations are still bearing fruit, and will undoubtedly help to maintain their future productivity.

To us, John was not only a mentor and friend, but also a family member who was loved and will be greatly missed by everyone.

